# Heteroatom-Rich Carbon Nanomaterials from Conjugated Polymers for Electrochemical Sensors

**DOI:** 10.3390/polym18172067

**Published:** 2026-08-25

**Authors:** Trong Danh Nguyen, Jun Seop Lee

**Affiliations:** Department of Materials Science and Engineering, College of Engineering, Gachon University, 1342 Seongnam-daero, Sujeong-gu, Seongnam-si 13120, Gyeonggi-do, Republic of Korea; ntdanh041@gachon.ac.kr

**Keywords:** conjugated polymers, elemental doping materials, carbon materials, electrochemical sensing

## Abstract

Tunable conductivity, heteroatom-rich composition, controllable morphology, and strong interfacial activity have resulted in carbon nanomaterials emerging as promising electrode modifiers for electrochemical sensors. As sources of sp2-rich carbon nanomaterials, conjugated polymers can be transformed through simple carbonization into carbon frameworks. Among them, polypyrrole- and polyaniline-derived carbon nanomaterials have been widely investigated for electrochemical sensing applications. In contrast, carbon nanomaterials derived from poly (3,4–ethylenedioxythiophene) have received comparatively limited attention, possibly due to the relatively high cost and established electrical conductivity of the polymer chain itself. The heteroatoms originally present in these polymers can be retained or transformed into active sites to promote electron transfer, analyte adsorption, and catalytic signal generation. This review summarizes recent progress in conjugated-polymer-derived carbon nanomaterials for electrochemical sensor applications, emphasizing precursor chemistry, morphology control, surface chemical regulation, metal and inorganic decoration, and sensing mechanisms. By clarifying the relationships among precursor structure, carbon framework, surface functionality, and electrochemical performance, this review offers guidance for designing sensitive and stable carbon-based sensing platforms.

## 1. Introduction

Electrochemical sensors have attracted sustained attention as practical analytical platforms, as they offer rapid response, high sensitivity, simple operation, low cost, and good compatibility with miniaturized devices [1,2]. These advantages make them suitable to detect environmental pollutants, biomolecules, food contaminants, pharmaceutical compounds, and disease-related markers [3]. In a typical electrochemical sensor, the target analyte interacts with the electrode surface through adsorption, recognition, or catalytic reaction, this interfacial event being converted into a measurable electrical signal [4]. Therefore, the sensing performance is strongly governed by the electrode-modifying material, which determines the accessible active sites, electron transfer kinetics, surface adsorption behavior, and stability of the sensing interface [5,6].

Due to their good electrical conductivity, chemical stability, broad potential window, tunable surface chemistry, and structural diversity, carbon-based nanomaterials are among the most widely investigated electrode modifiers [7]. However, the performance of the pristine carbon is not solely determined by electrical conductivity. Carbon surfaces often require functionalization, heteroatom doping, defect engineering, activation, or composite formation to create active sites, improve analyte adsorption, and enhance interfacial charge transfer [8,9]. In this context, polymer-derived carbon nanomaterials provide a versatile route to engineer carbon structures from molecular-designed precursors [10]. The selection of suitable polymer backbones, dopants, templates, and carbonization conditions makes it possible to control the carbon framework, porosity, heteroatom configuration, and surface activity [11]. Among different polymer precursors, conjugated polymers, such as polypyrrole (PPy), polyaniline (PANI), polythiophene (PT), and poly (3,4–ethylenedioxythiophene) (PEDOT), are particularly attractive options to prepare functional carbon nanomaterials [12]. Their π-conjugated backbones favor aromatization and the formation of sp^2^-rich carbon frameworks during pyrolysis, while their intrinsic heteroatoms can be retained or transformed into electrochemically active configurations [13]. Nitrogen species can contribute to electron transfer, defect generation, surface polarity, and analyte adsorption, whereas sulfur-containing structures can introduce additional charge redistribution and surface-active sites [14,15]. Moreover, their established polymerization routes allow the construction of various nanostructures, such as nanoparticles, nanotubes, nanofibers, hollow structures, and porous networks, which can be partially retained after the polymer nanomaterials undergoing carbonization [16,17].

Despite these advantages, several issues remain unresolved. The relationship between polymer precursor structure, carbonization pathway, heteroatom evolution, and final sensing performance has yet to be clearly established [18]. Many studies report enhanced sensitivity after doping, activation, or composite formation, but the individual contribution of each dopant, defect type, pore structure, or active site remains difficult to distinguish. In addition, although nitrogen-rich carbons have been widely explored, other components (e.g., sulfur-, phosphorus-, boron-, fluorine-, and multi-heteroatom)–doped systems remain less systematically compared [19]. Metal-containing conjugated-polymer-derived carbons also require clearer classification, because metals may act as coordination centers, graphitization catalysts, sacrificial templates, or active sensing sites [20,21].

**Figure 1:**
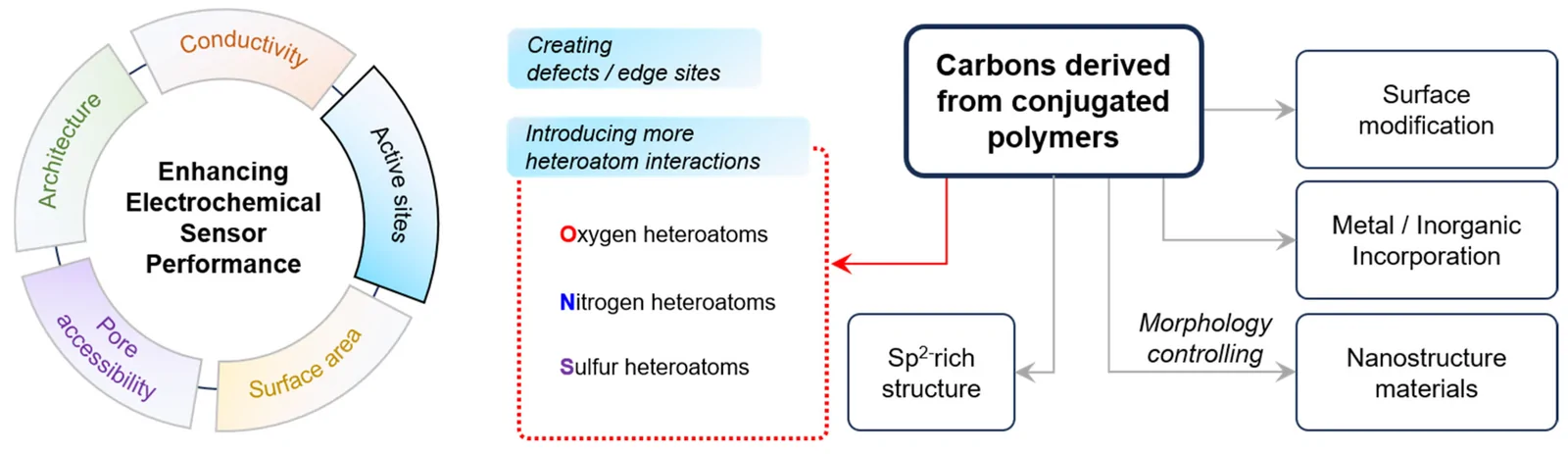
Roles and advantages of carbon materials derived from conjugated polymers in strategies to improve the performance of electrochemical sensors.

This review focuses on recent progress in carbon nanomaterials derived from conjugated polymers for electrochemical sensor applications (Figure 1). Particular attention is given to ring-containing conjugated polymer precursors, particularly PPy, PANI, PT, and PEDOT, including their precursor chemistry, carbonization behavior, heteroatom evolution, and morphology retention. Meanwhile, PA and non-conjugated polymers are included as reference systems for comparison of carbonization behavior. The review then addresses morphology control, surface chemical regulation beyond intrinsic heteroatoms, metal and inorganic decoration, and representative electrochemical sensor applications. By clarifying these structure–property–performance relationships, this review aims to guide the rational design of conjugated-polymer-derived carbon nanomaterials for sensitive, selective, and electrochemical sensing platforms.

## 2. Carbon Nanomaterials Derived from Polymer Precursors

Polymer-derived carbon nanomaterials have been widely studied, because polymer precursors can be designed pre-carbonization [22,23]. Their backbone chemistry, functional groups, heteroatom content, and molecular architecture strongly affect the carbonization pathway, carbon yield, heteroatom retention, porosity, defect density, and final carbon framework. During heat treatment, polymer precursors generally undergo stabilization, carbonization, and in some cases, graphitization [24]. Stabilization improves thermal stability and helps preserve the precursor morphology. Carbonization then converts the polymer into a carbonaceous framework through dehydrogenation, bond cleavage recombination, aromatization, and sp^2^ carbon domain formation [25]. Although graphitization can further improve structural ordering, it is not always necessary for electrochemical sensor applications [26,27]. The chemical structure of the polymer precursor is critical for efficient carbon formation. Polymers with aromatic, conjugated, or heteroatom-containing structures are generally more suited to forming carbonaceous frameworks [28]. In contrast, polymers that easily decompose or depolymerize during heating often show low char yields [29]. Therefore, precursor selection is an important starting point to design carbon nanomaterials for electrochemical sensing.

### 2.1. Aliphatic and Non-Conjugated Polymer Precursors

**Figure 2:**
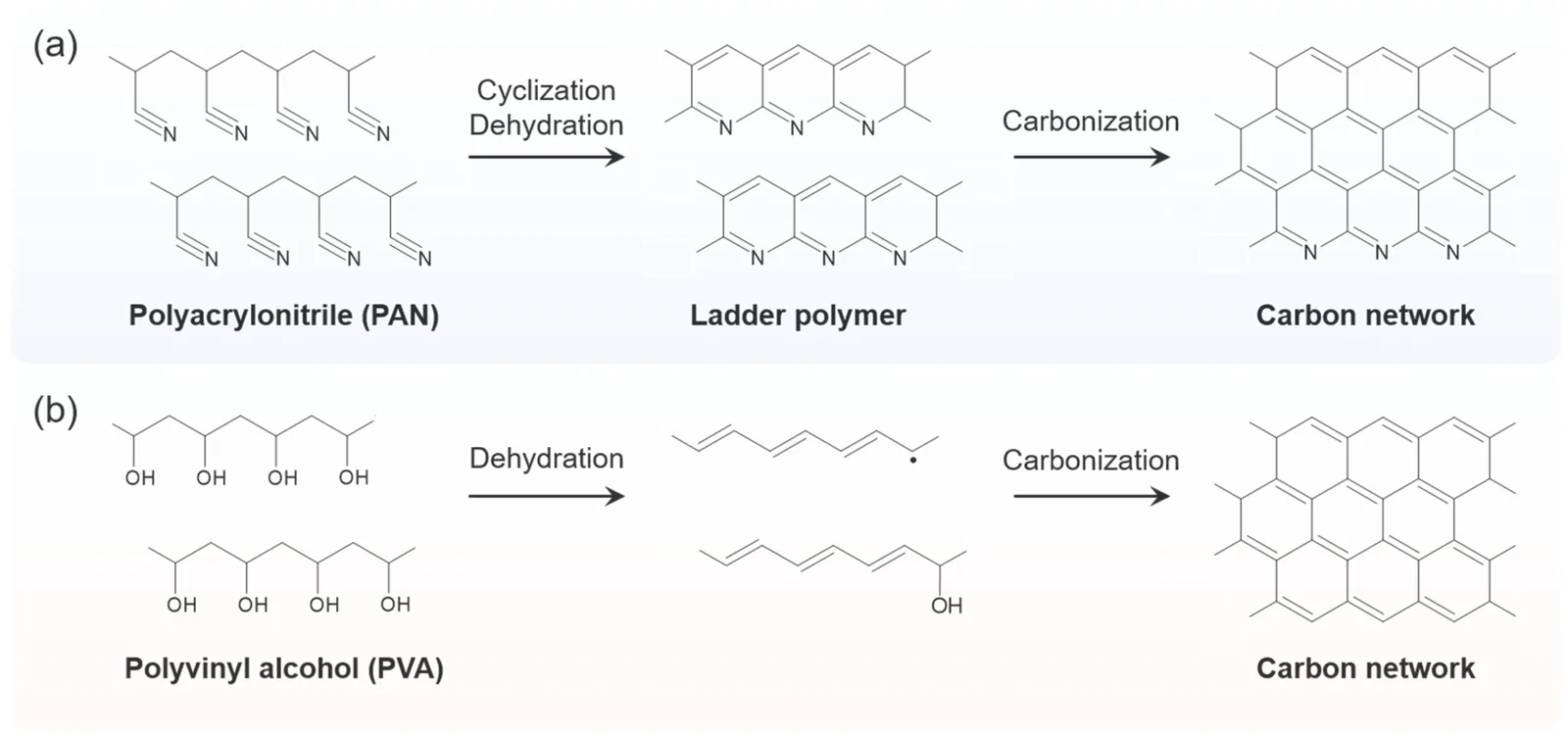
Carbonization mechanism of aliphatic polymer chains through heat treatment: (**a**) PAN, (**b**) PVA.

Aliphatic and non-conjugated polymers are useful reference systems to understand polymer-derived carbons. Representative examples include polyacrylonitrile (PAN) and poly (vinyl alcohol) (PVA) [30,31]. These polymers can be converted through stabilization and carbonization into carbon materials (Figure 2) [32,33,34]. In the stabilization stage, PAN mainly undergoes cyclization of nitrile groups to form a thermally stable ladder-like structure [35]. PVA can undergo dehydration, oxidation, and crosslinking [36,37]. Although the detailed reactions differ, stabilization commonly helps prevent severe melting, decomposition, or morphology collapse during carbonization. During carbonization, dehydrogenation, bond cleavage, and radical recombination promote aromatization and pore development [38,39], while increasing temperature generally enhances conductivity but can reduce heteroatom retention and surface functionality, requiring a balance among conductivity, defect density, and surface chemical activity [40].

PAN is a representative nitrogen-containing carbon precursor that offers relatively high carbon yield [41,42]. However, high total nitrogen content does not always guarantee high electrochemical activity [43,44]. During stabilization and carbonization, part of the nitrogen can be incorporated into the inner carbon framework, where these nitrogen atoms may not be easily accessible to the electrolyte or target analytes [45]. Thus, the type, location and accessibility of nitrogen species are of more consequence than the total nitrogen content alone [46]. This limitation provides the rationale to use conjugated polymer precursors. Conjugated polymers can combine carbon framework formation with intrinsic heteroatom sources that are more directly related to surface activity.

### 2.2. Linear Conjugated Polymer Precursors

**Figure 3:**
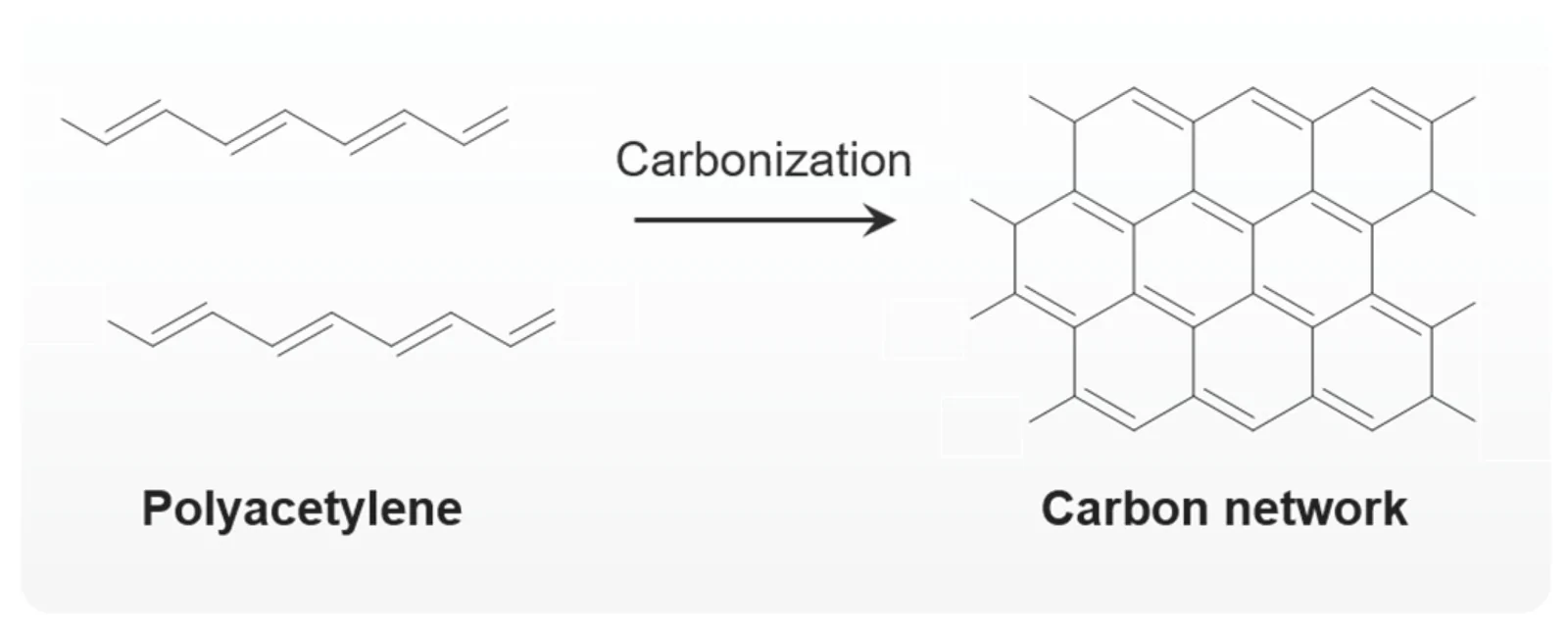
Carbonization mechanism of polyacetylene chains through heat treatment.

Polyacetylene (PA) is a representative linear conjugated polymer precursor, whose fully conjugated carbon backbone can promote dehydrogenation, aromatization, and the formation of sp^2^-rich carbon domains (Figure 3) [47,48]. Therefore, PA is useful as a model system to understand the carbonization of conjugated polymer backbones.

However, PA has important limitations for electrochemical sensor applications. Its chemical structure mainly contains carbon and hydrogen; therefore, intrinsic heteroatoms cannot be introduced into the carbon framework without additional dopants, catalysts, or post-treatment processes [49]. Its air and oxidation sensitivity also limit processing, storage, and practical use [50,51]. For these reasons, PA is better regarded as a conceptual bridge, rather than a major precursor for element-rich sensing carbons. While it shows that conjugation promotes sp^2^ carbon formation, it also shows that conjugation alone is insufficient for electrochemical sensing, where accessible heteroatoms and surface-active sites are also required [52].

### 2.3. Ring-Containing Conjugated Polymer Precursors

Ring-containing conjugated polymers are particularly suited to preparing element-rich carbon nanomaterials [53,54]. Representative examples include polypyrrole (PPy), polyaniline (PANI), polythiophene (PT), and poly (3,4–ethylenedioxythiophene) (PEDOT) [55,56,57]. These polymers contain aromatic or heteroaromatic structures within their backbones. Therefore, they can support aromatization and sp^2^ carbon formation during pyrolysis. Another important advantage is the presence of intrinsic heteroatoms. PPy and PANI contain nitrogen atoms, whereas PT and PEDOT contain sulfur atoms. After carbonization, these heteroatoms can be partially retained, or transformed into doped sites [58]. Such doped sites can improve surface polarity, charge distribution, analyte adsorption, and electron transfer behavior. Unlike many aliphatic polymer precursors, PPy and PANI do not always require a separate stabilization step, and can often be directly carbonized under an inert atmosphere [59]. Carbonization is commonly conducted at about (700–900) °C. In this temperature range, sufficient sp^2^ carbon domains can be formed, while useful nitrogen species can remain in the carbon framework.

#### 2.3.1. Nitrogen-Containing Conjugated Polymers: PPy and PANI

**Figure 4:**
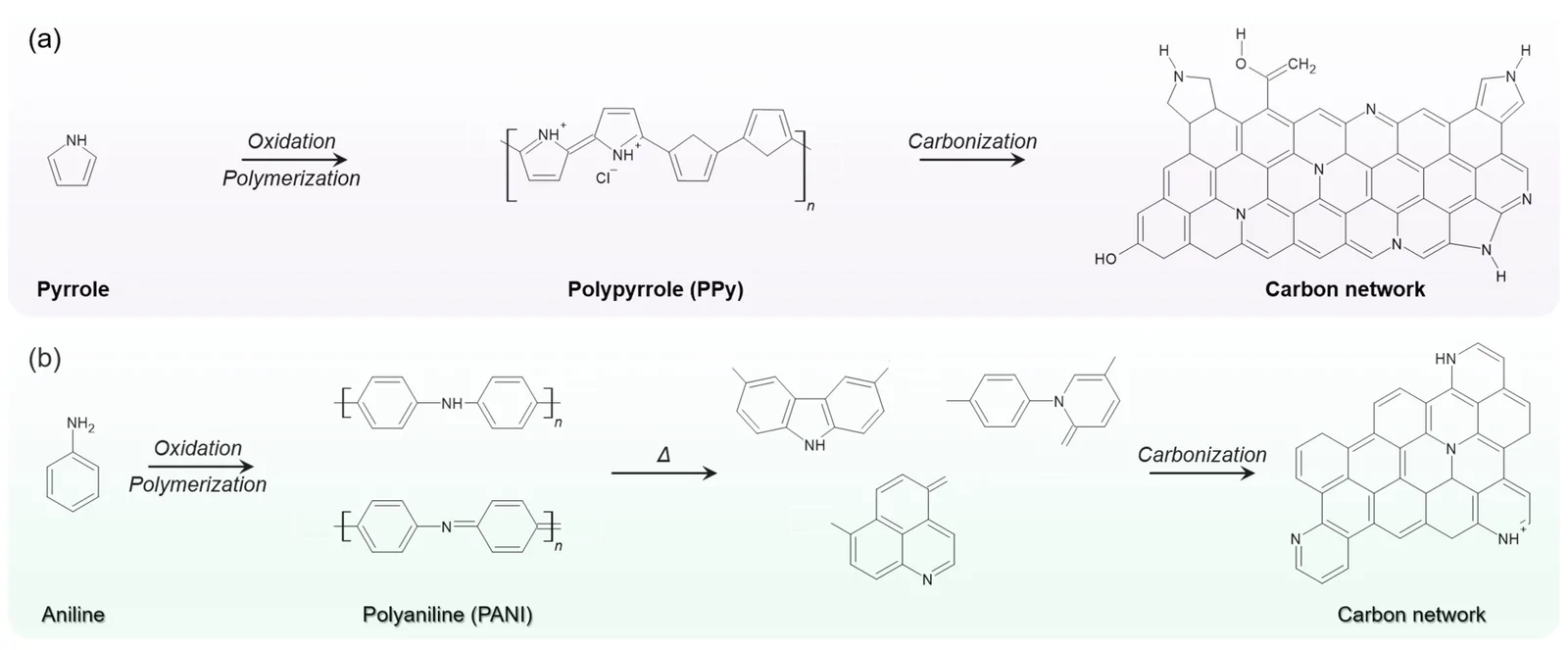
Chemical structure of conductive polymers and structural changes during the carbonization process: (**a**) polypyrrole, (**b**) polyaniline.

PPy and PANI have been widely investigated as nitrogen-containing carbon precursors for electrochemical sensors [60]. They can be synthesized relatively easily from pyrrole and aniline monomers [61,62]. Their polymerization routes also allow the formation of various nanostructures before carbonization [63]. These features make them practical precursors to prepare N-doped nanomaterials. The nitrogen atoms in PPy and PANI are present in different chemical environments: PPy contains nitrogen atoms within five-membered pyrrole rings, while PANI contains amine and imine nitrogen atoms between phenyl rings. These structural differences affect nitrogen retention, carbonization behavior, and the final nitrogen configuration in the carbon framework. For Ppy-derived carbon, the resulting materials commonly contain pyrrolic N, pyridinic N, and minor oxidized N species (Figure 4a) [64]. This is chemically reasonable, because the pyrrole ring acts as an intrinsic nitrogen source [65]. During carbonization, part of the pyrrolic nitrogen can be transformed into more thermally stable pyridinic or graphitic nitrogen [66]. However, carbonization temperature can decrease the total nitrogen content [67]. PANI-derived carbon shows a somewhat different behavior; the phenyl rings in PANI can promote the formation of sp^2^-rich carbon domains (Figure 4b), which can improve electrical conductivity post-carbonization [68,69]. However, the amine and imine nitrogen species can be removed more easily during pyrolysis through volatile nitrogen-containing species. Therefore, the final nitrogen content and configuration depend strongly on the carbonization temperature, holding time, atmosphere, activation method, morphology, and the presence of catalysts of metals [70]. PPy- and PANI-derived carbons are important, because their nitrogen species can be more accessible than those found in some conventional polymer-derived carbons. Although the total nitrogen content may sometimes be lower than that of PAN-derived carbon, the position and chemical configuration of nitrogen can be more favorable. Surface-exposed pyridinic, pyrrolic, and graphitic nitrogen sites can directly interact with electrolytes and target analytes; therefore, accessible nitrogen configuration is often more important than bulk nitrogen content [71].

**Figure 5:**
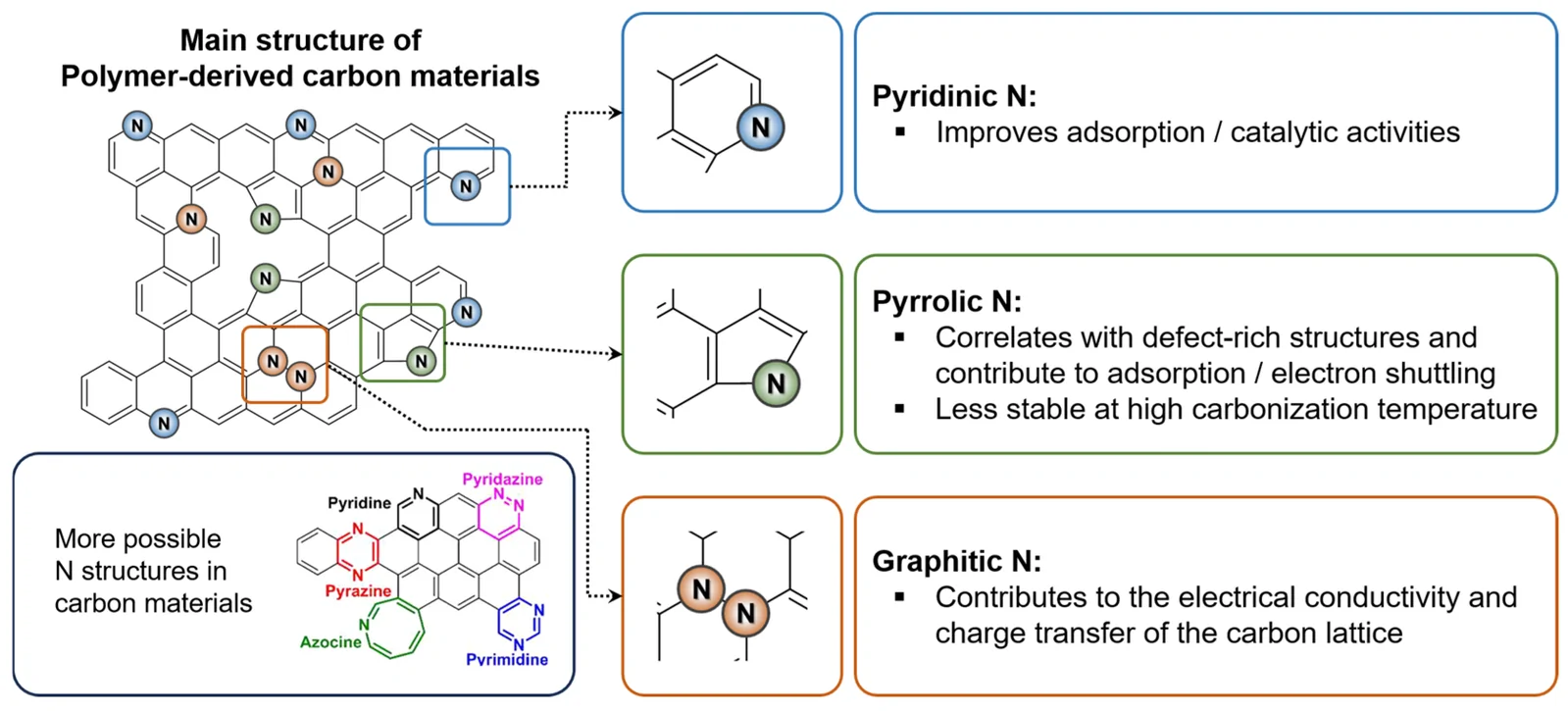
Types of N heteroatoms in carbon materials derived from polymer materials.

The main nitrogen configurations in these carbons have different functions (Figure 5). Pyridinic nitrogen is usually located at edges or defect sites of the carbon framework; this can induce local charge redistribution and generate electrochemically active sites [18,72,73]. Pyrrolic nitrogen is commonly associated with five-membered ring structures and defect-rich regions, and can improve surface polarity, wettability, and analyte adsorption [74]. Graphitic nitrogen substitutes carbon atoms within the sp^2^ lattice, which can improve electrical conductivity and promote charge transfer through the conjugated carbon network [75]. PPy and PANI are attractive not merely as carbon sources, but also as precursor-level nitrogen sources. Their carbonization can generate N-doped carbon frameworks with active surface chemistry. This feature is highly valuable for electrochemical sensors, where sensing performance is strongly influenced by surface adsorption, interfacial electron transfer, and catalytic signal generation.

#### 2.3.2. Sulfur-Containing Conjugated Polymers: PT and PEDOT

**Figure 6:**
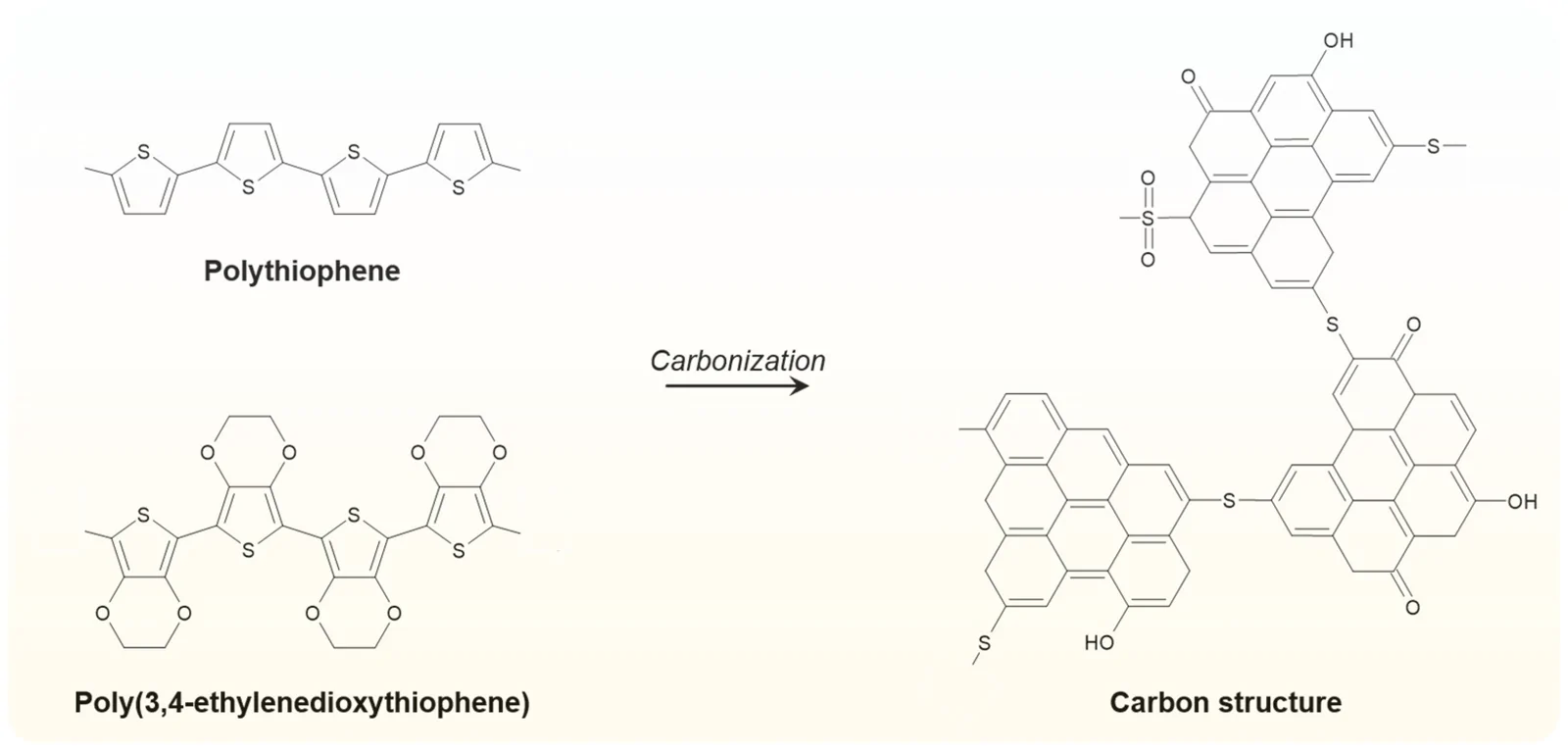
Chemical structure of polythiophene/poly (3,4–ethylenedioxythiophene), and structural changes during the carbonization process.

PT and PEDOT are sulfur-containing conjugated polymer precursors that, unlike PPy and PANI, are polymers that contain sulfur atoms in their heteroatomic structures. Therefore, their carbonization can potentially generate sulfur-doped carbon materials [76]. Sulfur species can modify the electronic structure of carbon and introduce additional surface-active sites (Figure 6) [77]. PEDOT is particularly interesting, because it is already a highly conductive polymer [78]. However, PEDOT-derived carbon nanomaterials have been less explored than PPy- and PANI-derived carbons. One reason is that the PEDOT already offers excellent electrical conductivity; therefore, carbonization may not always provide a clear advantage over the original polymer. In addition, many studies on sulfur-doped carbon materials have focused more on energy storage than on electrochemical sensing [79]. As a result, the use of PEDOT- or PT-derived carbon materials in electrochemical sensors remains relatively limited. Nevertheless, direct carbonization studies demonstrate that these polymers can retain sulfur within electrochemically active carbon frameworks. For example, carbonization and activation of polythiophene have produced S-containing porous carbons in which sulfur is retained predominantly as carbon-bonded species, while the resulting porous structure provides efficient ion-accessible electrochemical interfaces [80]. Similarly, PEDOT films can be directly converted into morphology-retaining carbonaceous films, with sulfur remaining detectable even after carbonization at temperatures up to 1100 °C [79]. Although these materials have mainly been investigated for electrochemical energy storage applications, their combination of retained sulfur species, conductive carbon domains, and accessible porous structures provides characteristics directly relevant to electrochemical sensing, including analyte adsorption and interfacial electron transfer. Nevertheless, sulfur-containing conjugated polymers still offer promise as precursors. Sulfur has a larger atomic radius than carbon, and can distort or polarize the carbon framework [76]. This effect can create defects, modify charge distribution, and strengthen interactions with target analytes. Therefore, although direct electrochemical sensing studies using carbonized PT and PEDOT remain scarce, the demonstrated retention of sulfur, conductive carbon framework, and precursor-derived morphology suggests that these materials represent an underexplored class of sensing electrode materials rather than merely general S-doped carbons. They can provide sulfur-containing carbon frameworks that complement nitrogen-doped carbons from PPy and PANI. Future studies may expand their use by combining sulfur doping with morphology control, activation, metal incorporation, or multi-heteroatom doping.

## 3. Morphological Control of Conjugated-Polymer-Derived Carbon Nanomaterials

Morphology is a key factor that determines the electrochemical sensing performance of carbon nanomaterials [81,82]. Even with similar chemical composition, sensing behavior can vary depending on the surface area, pore accessibility, diffusion pathway, and electron transport network [83]. Therefore, controlling the morphology of the conjugated polymer precursor is important in the design of high-performance carbon-based electrodes. In polymer-derived carbon materials, morphology is usually determined pre-carbonization [84]. This is particularly important for conjugated polymers, such as PPy, PANI, and PEDOT. Their rigid and highly conjugated backbones often limit post-polymerization processing. Thus, the desired nanostructures are commonly formed during polymerization or precursor assembly [85,86,87,88,89,90].

### 3.1. Morphological Design of Conjugated Polymer Precursors

**Figure 7:**
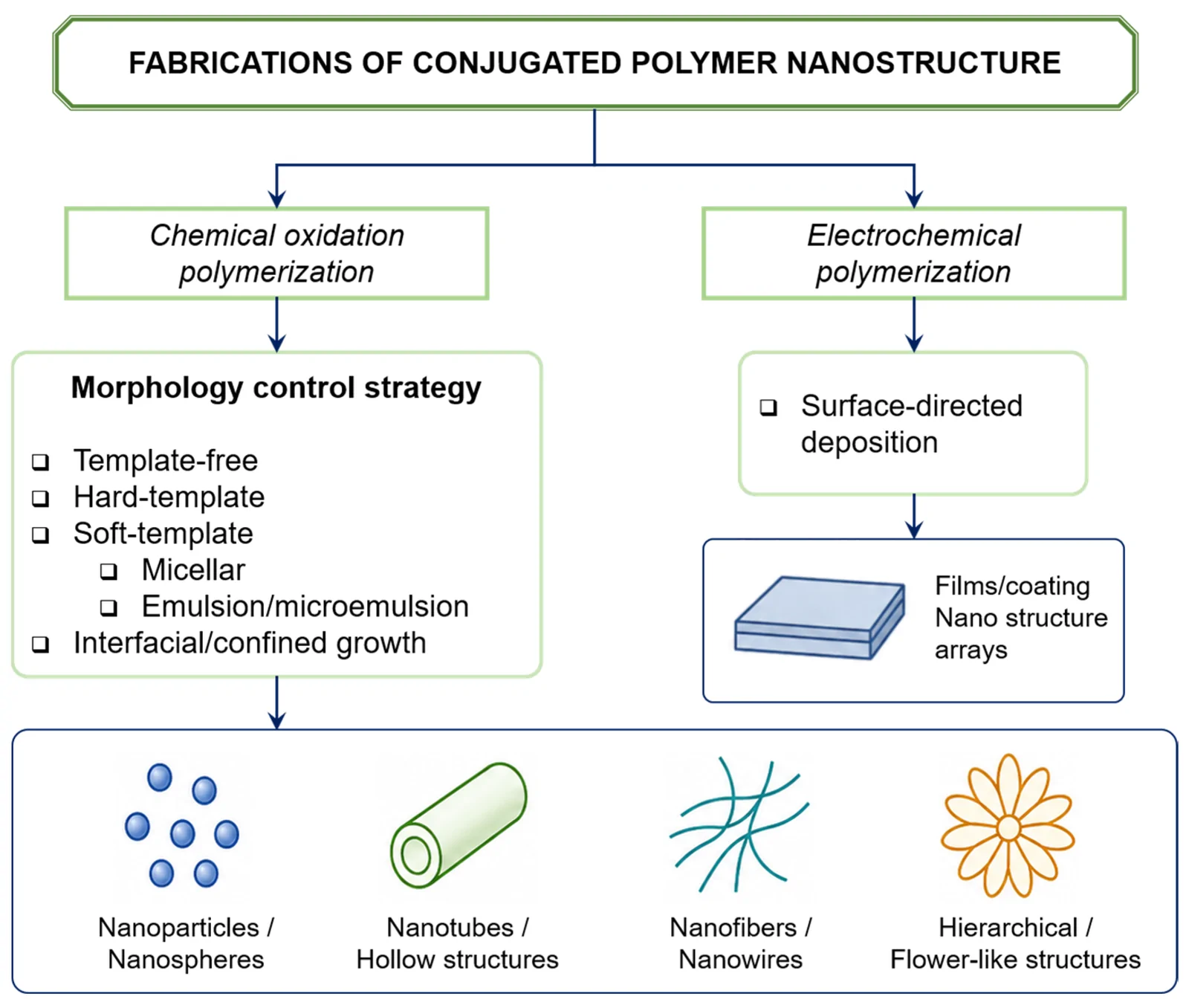
Conjugated polymer nanomaterials of various morphologies formed using diverse manufacturing principles.

Several approaches have been developed to prepare conjugated polymer nanostructures with controlled morphology [91,92]. These include soft-template, hard-template, template-free, heterogeneous polymerization, interfacial polymerization, and confined polymerization methods (Figure 7). Using these methods, pyrrole, aniline, and 3,4–ethylenedioxythiophene monomers can be converted into PPy, PANI, and PEDOT nanomaterials with various morphologies [93,94]. Soft-template methods use dynamic assemblies as nanoscale reactors. Micelles, emulsion droplets, mini-emulsion droplets, micro-emulsion domains, and vesicles can guide the formation of particles, capsules, hollow structures, and other nanostructures [95,96]. In contrast, hard-template methods use rigid structures, such as porous membranes, silica particles, or anodic aluminum oxide templates [97,98]. While these methods provide good morphological control, template removal is often required. Template-free methods rely on polymer insolubility, nucleation, precipitation, dispersion, and colloidal stabilization [99]. These approaches are relatively simple, but precise control over size and shape can be challenging. Interfacial and confined polymerization methods localize polymer formation at the phase boundaries of interior confined spaces; this strategy is useful to produce shells, hollow structures, capsules, and anisotropic nanostructures [100]. Some examples of carbonizations and structural characteristics of conjugated-polymer-derived carbon materials are demonstrated in Table 1.

### 3.2. Morphological Retention During Carbonization

One advantage of conjugated polymer nanostructures is that, after carbonization, their morphology can often be partially retained [108]. Although during pyrolysis, shrinkage and densification commonly occur, severe shape collapse can be reduced when the precursor structure is of adequate stability [109]. This allows the morphology of PPy, PANI, or PEDOT precursors to be transferred into the final carbon nanomaterials. Morphology retention is valuable for electrochemical sensors. Carbonization can convert conjugated polymer nanostructures into conductive carbon frameworks, while maintaining nanoscale geometry. In consequence, the final carbon materials can provide high surface area accessible pores, and short ion-diffusion pathways. These features enhance the interaction between the electrode surface and target analytes [110,111]. However, morphology retention is not always complete. During carbonization, polymer nanostructures can shrink, collapse, or become brittle. Hollow structure and nanotubes may suffer from wall shrinkage or channel blockage. After pyrolysis, nanofibers can become denser or more fragile. Therefore, the precursor morphology, wall thickness, carbonization temperature, heating rate, and atmosphere should all be carefully controlled [112].

### 3.3. Morphology-Dependent Sensing Characteristics

The morphology of conjugated-polymer-derived carbon nanomaterials strongly influences electrochemical sensing performance by controlling surface exposure, mass transport, and electron transfer pathways [113]. Although nanoparticles provide large surface-to-volume ratios and short diffusion lengths, which are useful for rapid sensing responses, their aggregation can reduce the effective electroactive area, and increase interparticle resistance [114]. One-dimensional structures, such as nanotubes and nanofibers, offer more continuous pathways for charge transport [115,116]. Nanotubes provide inner and outer surfaces, as well as hollow channels that facilitate electrolyte penetration and analyte diffusion [117]. Nanofibers form conductive networks with fewer junctions, to improve mechanical integrity and electron collection [118]. However, nanotube collapse, channel blockage, fiber densification, or limited electrolyte penetration can reduce the use of active sites post-carbonization [119]. Hierarchical structures, including porous networks and nanoflower-like morphologies, can combine high surface area with improved mass transport. Because these structures expose abundant edge sites and active surfaces, they are attractive for high-sensitivity sensing; however, their reproducibility and structural stability during post-treatment or carbonization remain challenging [120,121].

## 4. Surface Chemical Regulations Beyond Intrinsic Heteroatoms

After the carbon framework is formed from a conjugated polymer precursor, sensing performance can be further improved by regulating the surface chemistry [122]. This section focuses on surface functional groups and additional dopants beyond the intrinsic nitrogen or sulfur atoms supplied by the polymer backbone [123]. These strategies are important, because electrochemical sensing mainly occurs at the electrode–electrolyte interface [124]. Surface chemical regulation can control wettability, adsorption strength, surface charge, defect density, and interfacial electron transfer behavior [125]. However, excessive modification can damage the conductive sp^2^ carbon network; therefore, surface activation should be balanced with electrical conductivity and structural stability [126,127].

### 4.1. Oxygen-Containing Surface Functional Groups and Electrochemical Interfaces

Oxygen-containing functional groups are commonly introduced during activation, oxidation, plasma treatment, or mild chemical treatment [128]. These groups include hydroxyl, carbonyl, carboxyl, epoxy, and quinone-like structures [129]. They improve the hydrophilicity of carbon electrodes, and enhance electrode–electrolyte contact [130]. Improved wettability allows electrolytes and target analytes to more effectively access the electrode surface [131]. Oxygen-containing groups can also provide adsorption sites for polar analytes. They can also serve as anchoring sites for metal nanoparticles, enzymes, or molecular recognition units [132,133]. Some oxygen functional groups can participate in surface redox reactions. This may contribute to signal amplification, or enhance apparent electrochemical activity [134]. However, excessive oxygen functionalization can disrupt the sp^2^ carbon network, increase background current, and reduce long-term stability; therefore, oxygen functionalization should be carefully controlled [135].

### 4.2. Additional Heteroatom Incorporation Beyond the Intrinsic N and S

Other heteroatoms can be introduced to further tune the surface chemistry of conjugated-polymer-derived carbons [136,137]. Phosphorus, boron, and fluorine are representative examples: these elements modify the electronic structures, surface polarity, and local charge distribution of carbon nanomaterials. Because of its large atomic size and different electronegativity compared to carbon, phosphorus-doping can generate defect sites [138]. These defects can increase surface polarity and provide adsorption sites for ionic or polar analytes [139]. Boron-doping introduces electron-deficient or Lewis–electrochemical oxidation reactions [140,141]. Fluorine-doping can strongly polarize the carbon surface because of the high electronegativity of fluorine; this can help tune surface charge and selectivity [142]. But through strong C−F bonding, excessive fluorination may reduce conductivity [143]. Therefore, fluorine incorporation requires careful control. Multi-heteroatom regulation can provide synergistic effects. For example, combining phosphorus or boron with intrinsic heteroatom-containing carbon can tune both charge distribution and adsorption behavior; however, excessive or poorly controlled doping can cause unstable bonding, dopant loss, pore blockage, or reduced conductivity [144,145].

## 5. Metal and Inorganic Decoration of Carbon Nanomaterials

**Figure 8:**
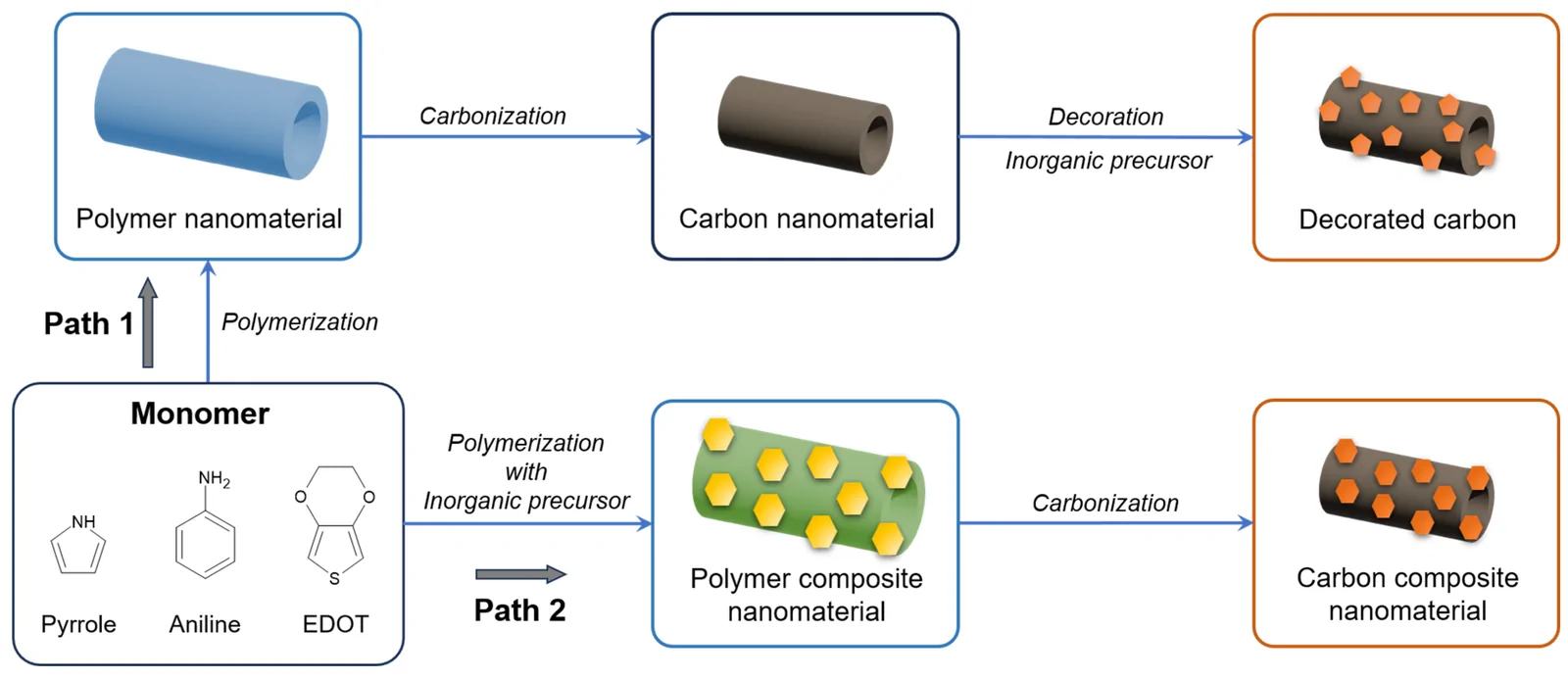
Various methods and mechanisms to fabricate carbon nanomaterials incorporated with metals or metal oxides.

Metal and inorganic components can provide catalytic and recognition functions that are difficult to achieve via the carbon framework alone [146,147]. In electrochemical sensors, these components can promote analyte oxidation or reduction, accelerate charge transfer, and improve selectivity. Therefore, metal and inorganic decoration is an effective strategy to extend the function of conjugated-polymer-derived carbon nanomaterials (Figure 8). Conjugated-polymer-derived carbons are suitable platforms for metal incorporation, because PPy and PANI can act as nitrogen-containing coordination matrices pre-carbonization [148,149]. Their nitrogen sites can bind metal ions, and distribute them within the polymer precursor [150]. After pyrolysis, these interactions can be converted into metal–nitrogen sites, embedded nanoparticles, or strongly coupled metal/carbon interfaces [151]. This precursor-level interaction presents an important advantage over simple post-carbonization mixing. When metals are introduced after carbonization, they are often attached only to the external carbon surface, which can lead to weak anchoring, aggregation, and limited electronic coupling. In contrast, metal incorporation pre-carbonization can generate more integrated metal/carbon structures.

### 5.1. Pre-Carbonization Metal Incorporation

Pre-carbonization metal incorporation introduces metal species into the conjugated polymer precursor before pyrolysis. Metal salts, metal complexes, metal-containing oxidants, or metal-based templates can be incorporated during, or after, pyrrole or aniline polymerization [152,153,154]. The purpose is to distribute metal species within the polymer matrix, and promote interactions with nitrogen-containing sites. Transition metal ions, such as Fe, Co, Ni, Cu, Mn, and Zn, can interact with lone-pair electrons in PPy or PANI [155]. Thus, the conjugated polymer serves not merely as a carbon source, but also as a ligand-like scaffold. During thermal conversion, this scaffold helps immobilize metal species and suppress aggregation. This strategy is useful for electrochemical sensing, because it integrates conductive carbon domains and catalytic metal sites within, or on, the framework. The carbon matrix supports charge transport and analyte adsorption. The incorporated metals can provide catalytic activity, promote redox reactions, or improve operational stability. However, metal loading must be carefully controlled; excessive metal content can cause nanoparticle aggregation, pore blockage, or partial destruction of the precursor morphology. The type of metal precursor, metal-to-monomer ratio, polymerization condition, and carbonization atmosphere strongly affect the final active site structure [156,157].

### 5.2. In Situ Transformation During Carbonization

Although metal species are introduced before pyrolysis, their final active forms are usually generated during carbonization [158,159]. Under high temperature, the polymer precursor is converted into a doped carbon framework. At the same time, the metal species can be reduced, oxidized, coordinated, crystallized, or transformed into metal-containing compounds [160]. One important product is the metal–nitrogen coordination site, often described as M−N_x_, or M−N−C [161]. These sites can form when metal ions interact with nitrogen atoms retained in the carbon matrix during pyrolysis. M−N_x_ sites can act as catalytic centers for electrochemical oxidation or reduction reactions. Metal species can also be transformed into metallic nanoparticles, metal oxides, sulfides, phosphides, carbides, or nitrides [162]. The final phase depends on precursor composition and carbonization atmosphere. Surface-exposed species are especially important, because they can directly contact the electrolyte and target analytes [163]. In situ metal transformation can also affect the carbon framework itself. Transition metals, such as Fe, Co, and Ni, can promote the formation of more graphitic domains, which can improve electrical conductivity and electron transfer kinetics [164]. Metal salts may also support pore generation and defect formation during pyrolysis [165]. These effects can work synergistically in electrochemical sensors. Metal sites provide catalytic activity, while the carbon framework provides conductive pathways. Defects and pores expose more active centers, and improve electrolyte penetration. Strong metal–carbon coupling can also reduce metal leaching during repeated measurements [166]. Nevertheless, in situ transformation is complex, and can be difficult to control. The same precursor can produce different metal species depending on temperature, atmosphere, heating rate, and the chemical environment of the polymer. For example, at low loading, metal ions may form isolated M−N_x_ sites, but at higher loading, they may aggregate into nanoparticles [167]. Excessive graphitization may reduce defect density, while excessive defect formation may decrease conductivity. Therefore, the design of in situ transformed PPy/PANI-derived carbon requires a balance between metal site formation, carbon conductivity, porosity, and structural stability. In electrochemical sensing, the best performance is usually obtained when the metal species are well dispersed, electronically coupled with N-doped carbon, and sufficiently exposed to interact with the target analyte.

### 5.3. Metal Oxide and Inorganic Interfaces

Metal oxide and other inorganic components can further expand the function of conjugated-polymer-derived carbons [168]. Metal oxide can provide redox-active surfaces, catalytic sites, and analyte-specific adsorption sites [169]. When strongly coupled with carbon, the carbon framework can compensate for the limited conductivity of many inorganic materials [170]. MOF-derived or oxide-forming precursors are useful to generate such hybrid interfaces [171]. A metal-containing framework can act as a template or metal source, while the conjugated polymer coating provides a carbon-forming layer. After carbonization, the original inorganic structure may be retained, partially transformed, or converted into a metal oxide/carbon interface [172]. Even when the original template is not preserved, in situ transformation can still be valuable. During polymer carbonization, metal–organic precursors can be converted into metal oxide species. The resulting metal oxide/carbon interfaces can enhance catalytic sensing reactions and improve analyte adsorption [173,174]. After carbonization, inorganic components can also be introduced through physical mixing or surface decoration. This approach is simple, and useful to prepare composites. However, the interaction between carbon and inorganic components is usually weaker than that formed by pre-carbonization incorporation; weak coupling can reduce charge transfer efficiency and long-term stability [175].

## 6. Electrochemical Sensor Applications

Conjugated-polymer-derived carbon nanomaterials have been applied to various electrochemical sensors [176]. Their sensing performance is determined by the precursor chemistry, morphology, surface functionality, and metal or inorganic decoration [177,178]. Therefore, this section summarizes representative applications according to the dominant material design strategy.

### 6.1. Sensors Based on Intrinsic Carbon Structure and Morphology

The most basic application of conjugated polymer-based carbons is their use as conductive electrode modifiers. PPy- and PANI-derived carbons are representative examples, because they provide N-containing carbon frameworks post-carbonization [179,180]. Their intrinsic carbon structure can support charge transfer, while their nanostructure can increase the accessible electrode surface. Morphology is important in these systems: porous, tubular, fibrous, or nanoparticulate carbons can shorten diffusion pathways, and expose more active sites [181,182]. In this case, the porous structure improves analyte access, while the carbonized PANI framework supports electrochemical signal generation. Ppy-derived carbon nanotubes also show the importance of carbonization-controlled structure. Pyrolysis temperature can affect surface area, heteroatom retention, pore formation, and electrochemical activity. Higher temperatures may improve conductivity and porosity, but they can also reduce useful heteroatom content [183]. Thus, sensor performance requires a balance among carbon structure, surface area, and retained active sites.

**Figure 9:**
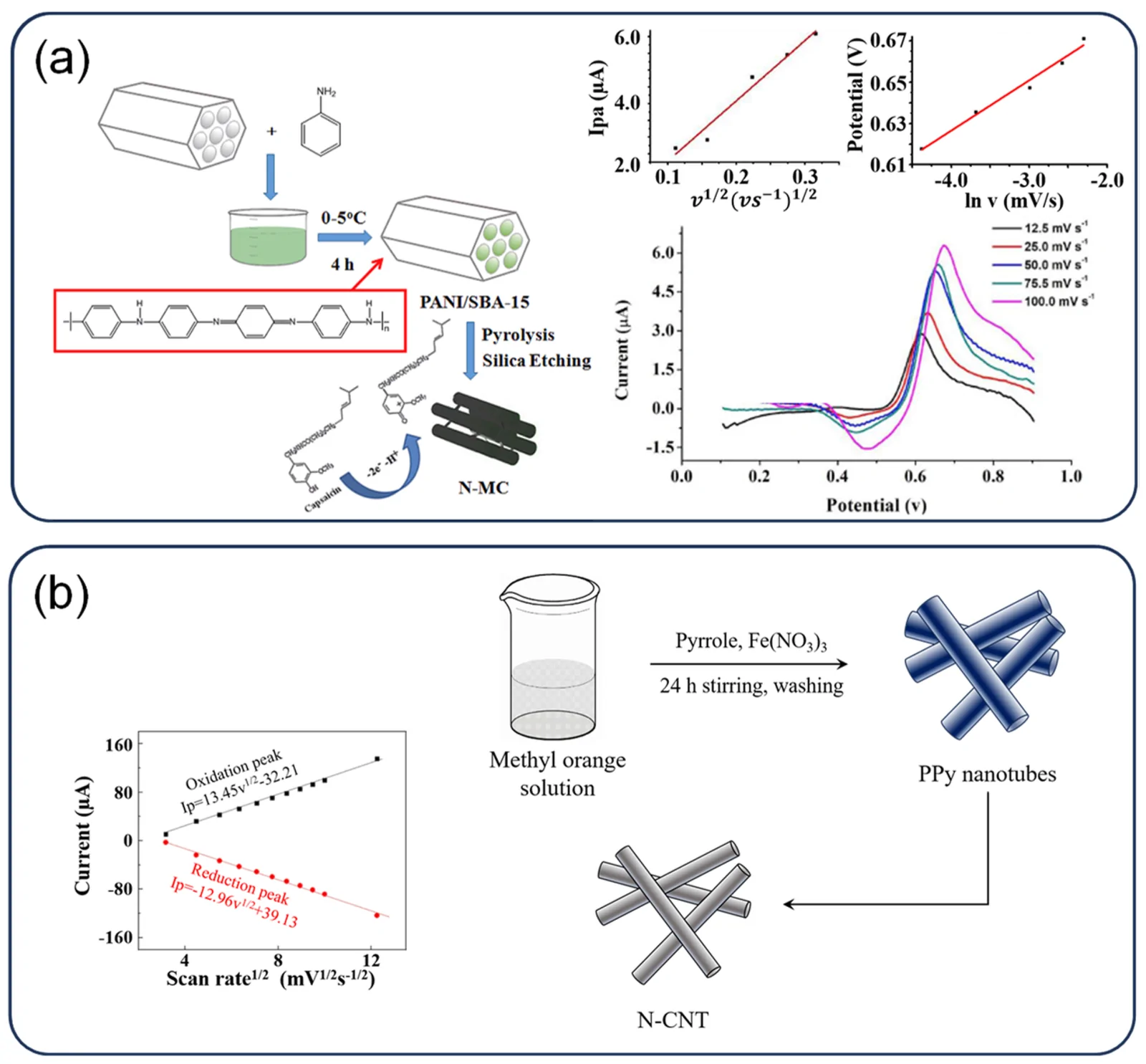
(**a**) Nitrogen-doped mesoporous carbon, which was used as an electrochemical sensing material for capsaicin detection, prepared by the direct carbonization of polyaniline [184]. Copyright 2019 @ Elsevier. (**b**) Polypyrrole-derived carbon nanotubes prepared by carbonizing polypyrrole nanotubes for dopamine detection [185].

Figure 9a shows an example in Temcheon et al. (2019) of the performance of PANI-derived carbon in electrochemical sensing, targeting the detection of capsaicin [184]. PANI nanomaterials were synthesized using mesoporous silica SBA−15 as a hard template, carbonized, and used without additional functional modification. The carbon material was subjected to mild alkaline treatment in 1 M NaOH at 100 °C for 6 h to remove the SBA−15 template, which can also be considered a surface-cleaning or mild surface activation process, rather than conventional high-temperature chemical activation. Thus, this work provides a useful view of the intrinsic capability of PANI-derived N-doped mesoporous carbon for electrochemical sensor applications. Wang et al. (2022) systematically investigated the changes in surface area and heteroatom concentration of Ppy-derived carbon nanotubes prepared at different pyrolysis temperatures (Figure 9b) [185]. Although the results still suggest that (700–800) °C remained a suitable temperature range, higher calcination temperatures caused greater heteroatom loss. Interestingly, the authors also stated that higher temperature facilitated the formation of porous structures, and that carbon nanotubes generated at 800 °C supported good electrochemical activity, as proven by cyclic voltammetry. However, further investigation of the sensing performance is still needed, because heteroatom species, surface chemistry, and pore structure are important for electrode performance in electrochemical sensors.

### 6.2. Sensors Enhanced by Surface Chemical Regulation

Surface chemical regulation is useful for analytes that require adsorption, preconcentration, or selective interfacial interaction [186]. Oxygen-containing groups, additional heteroatoms, and defects can improve wettability and electrode–electrolyte contact. These features are important for detecting polar organic molecules, biomolecules, and heavy metal ions [187]. For neurotransmitters and biomolecules, such as dopamine, serotonin, and nicotine, fast electron transfer and selectivity are required [188]. Heteroatom-rich carbon surfaces can promote adsorption through electrostatic interaction, hydrogen bonding, π–π interaction, or defect-mediated interaction. Because of their small size and abundant surface groups, carbon quantum dots or doped carbon nanostructures can further improve sensitivity [189,190]. Heavy metal ion detection also benefits from surface-regulated carbon materials. Cd (II), Pb (II), Hg (II), and related ions require electrode surfaces that can accumulate ions prior to electrochemical stripping or redox conversion [191,192]. Surface oxygen groups and additional heteroatom-containing sites can act as binding or coordination sites. Therefore, surface chemistry directly affects ion-preconcentration and analytical sensitivity [193]. Surface-regulated carbons are also useful for food and environmental contaminants. Phenolic compounds, nitroaromatic pollutants, and pharmaceutical residues can interact with polar or defect-rich carbon surfaces [194]. However, excessive surface oxidation or unstable functionalization may reduce conductivity and long-term stability.

**Figure 10:**
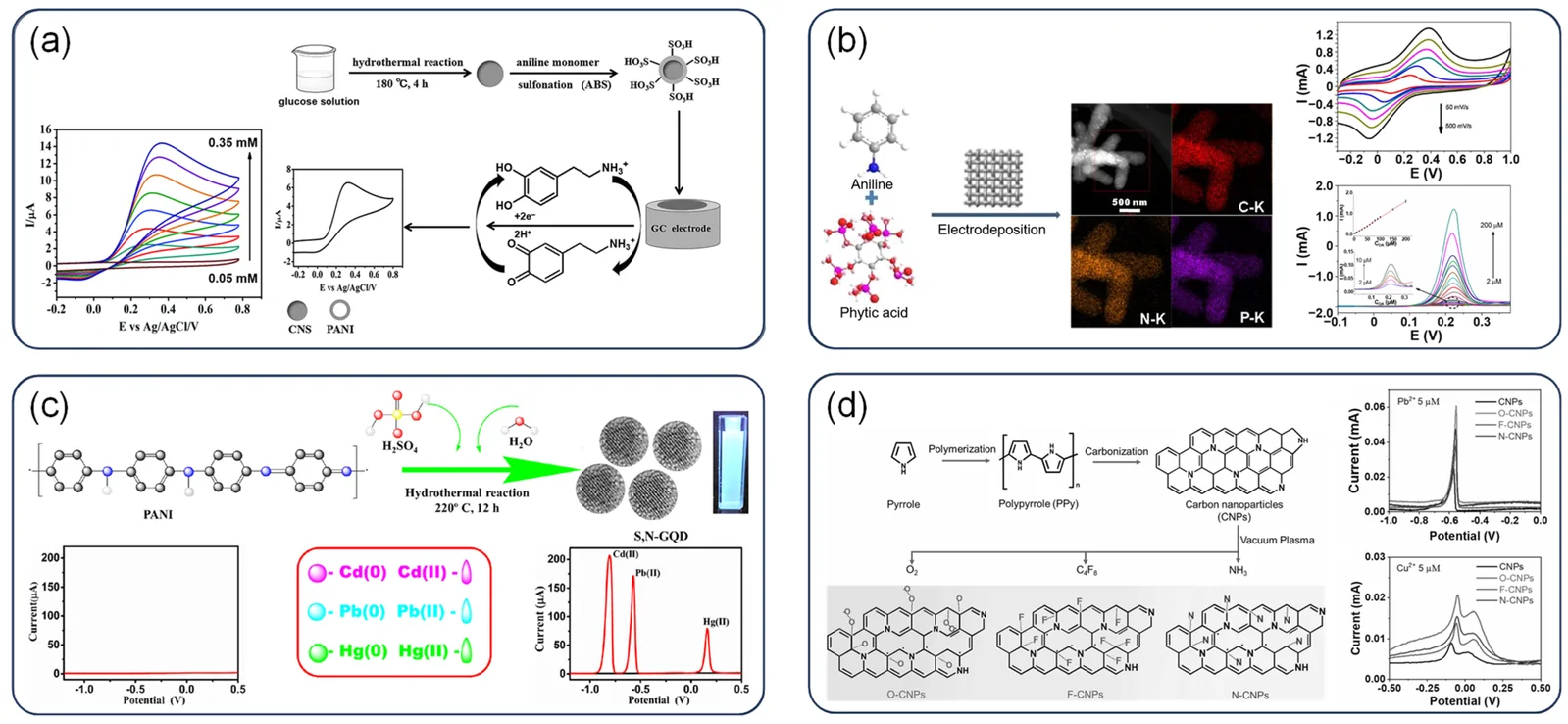
(**a**) Sulfonated polyaniline-decorated carbon nanospheres prepared by coating carbon nanospheres with sulfonated polyaniline, and applied for electrochemical dopamine detection [195]. Copyright 2017 @ Royal Society of Chemistry. (**b**) A flexible three-dimensional N,P-co-doped carbon network prepared by the electropolymerization of aniline/phytic acid on carbon cloth, followed by carbonization for the detection of dopamine [196]. Copyright 2017 @ Elsevier. (**c**) Sulfur and nitrogen co-doped graphene quantum dots prepared from polyaniline using sulfuric-acid-assisted treatment for the simultaneous electrochemical detection of Cd (II), Pb (II), and Hg (II) [197]. Copyright 2023 @ American Chemical Society. (**d**) Plasma-functionalized carbon nanoparticles prepared via vacuum plasma treatment of polypyrrole-derived carbon nanoparticles for the square-wave voltammetric detection of Pb (II) and Cu (II) [198].

**Figure 11:**
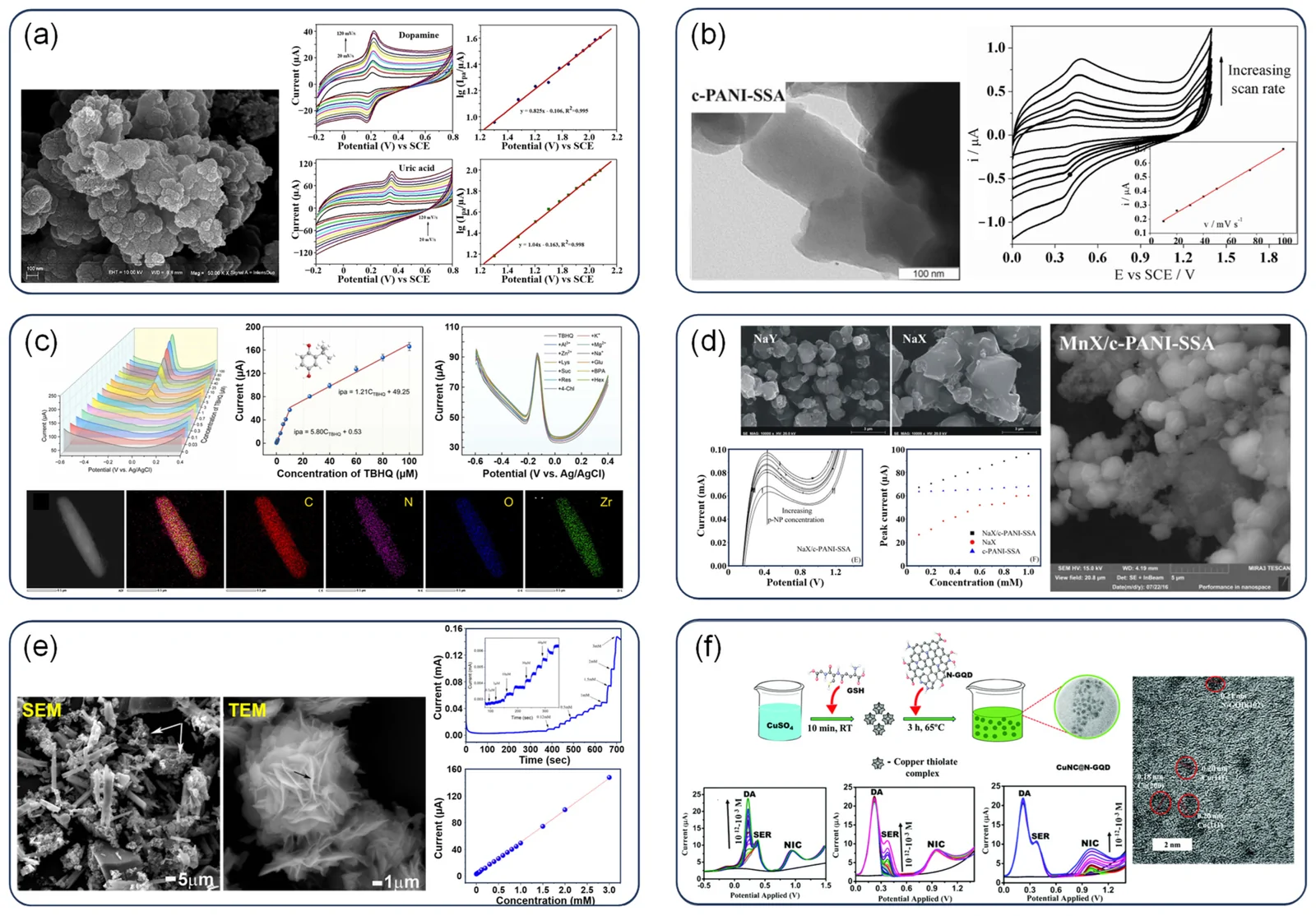
(**a**) Nitrogen-doped carbon-supported iron nanocomposite was prepared by pyrolyzing a FeCl_3_/polypyrrole precursor, and applied to the simultaneous electrochemical detection of dopamine and uric acid [204]. Copyright 2022 @ Elsevier. (**b**) The incorporation of HPW−BEA hybrid materials in carbonized polyaniline for electrochemical sensing of phenolic compounds in environmental water samples [204]. Copyright 2018 @ Elsevier. (**c**) MOF−545@PANI−800 prepared by the in situ oxidative polymerization of polyaniline on MOF−545, followed by carbonization for tert–butylhydroquinone detection [205]. Copyright 2022 @ Elsevier. (**d**) Composite zeolite/carbonized polyaniline for the electrochemical sensing of p–nitrophenol and related phenolic compounds in aqueous media [206]. Copyright 2016 @ Elsevier. (**e**) Flower-like C,N-doped NiO nanocomposites prepared by the calcination of Ni–MOF/polyaniline precursor for glucose detection [207]. Copyright 2021 @ Springer Nature. (**f**) CuNC@N−GQDs were prepared by synthesizing copper nanoclusters on nitrogen-doped graphene quantum dots using glutathione as an additional capping agent for the electrochemical detection of dopamine, serotonin, and nicotine [208]. Copyright 2022 @ Royal Society of Chemistry.

There are also cases where heteroatoms can be introduced into carbon materials through residual dopants, initiators, oxidants, or templates, besides those originating from the polymer structure (such as nitrogen atoms from PPy and PANI). The fabrication of nanomaterials based on PPy, aniline, or EDOT often requires templates, dopants, or oxidizing agents, which can be difficult to completely remove. Instead of viewing these remaining species simply as impurities, some studies have made use of them as sources of additional heteroatoms. For example, Fu et al. (2017) utilized sulfur from m–aminobenzenesulfonic acid as a sulfur source for carbon nanoparticles, as shown in Figure 10a [195]. Although the sensing performance of the resulting material was reported to be good, this concept is relatively old, and the quantity of additional heteroatoms is usually limited, especially after carbonization. Nevertheless, it is important to note that not all residual species in polymer precursors are harmful; if properly controlled, they can serve as heteroatom sources that improve the performance of carbon materials in electrochemical sensing. For other heteroatoms to be distributed evenly, their sources also need to be uniformly incorporated into the polymer precursor. One potential method is to introduce heteroatom sources during polymerization, thereby trapping them within the polymer network. Figure 11b shows the strategy proposed by Zhao et al. (2017), which exemplifies this approach [196]. The authors mixed phytic acid into an aniline-containing electrolyte for aniline electropolymerization, producing a PANI/phytic acid layer on carbon cloth; after carbonization, N,P-co-doped carbon materials were obtained, and used for dopamine detection. The drawback of this method is that, because electropolymerization is carried out directly on the surface of carbon cloth, the product quantity is low. However, in the field of electrochemical sensors, this is not a serious limitation, as only a small amount of active electrode material is usually required. On the other hand, Saisree et al. (2023) used sulfuric acid during the hydrothermal carbonization of PANI, as shown in Figure 10c [197]. In this process, sulfuric acid served not just as an acidic medium, but also as a sulfur doping source. In consequence, the obtained sulfur and nitrogen co-doped graphene quantum dots carried sulfur-containing species in their structure, thus allowing the material to show improved performance in the detection of heavy metal ions. The reported sensitivity values for Cd (II), Pb (II), and Hg (II) were (12, 13, and 5) μA·μM^−1^·cm^−2^, respectively, while the limit of detection values were (1, 10, and 1) pM, respectively, demonstrating the potential of sulfur co-doping to improve the electrochemical sensing performance of PANI-derived quantum dots.

While chemical modification and activation have been used for many years, plasma treatment is a surface modification method that offers high potential. Depending on the type of gas used during plasma exposure, different surface functional groups or heteroatoms can be introduced into carbon materials. Plasma treatment can also increase surface energy, improve wettability, remove surface contaminants, and create defect sites, all of which are useful for electrochemical sensing. However, this method also has limitations, because many parameters need to be carefully controlled, including gas type, pressure, treatment time, plasma power, and the distance between the plasma source and sample. Figure 10d shows a representative example of plasma treatment being used to introduce additional heteroatom-containing functional groups onto carbonized materials by Phan et al. (2022) [198]. The results indicated that the plasma-treated carbon materials showed improved electrochemical sensor performance, compared to the untreated materials. This improvement can be attributed to the modified surface chemistry, enhanced wettability, and increased number of electroactive sites after plasma treatment.

### 6.3. Sensors Enhanced by Metal and Inorganic Decoration

Some analytes require catalytic activity that carbon, by itself, cannot sufficiently provide [199]. In these cases, metal or inorganic decoration can introduce active sites for electrochemical oxidation or reduction. Conjugated-polymer-derived carbons are useful supports by providing conductive pathways and anchoring sites for metal-containing species [200]. Nitroaromatic compounds, such as 4–nitrophenol, can benefit from catalytic carbon/metal interfaces, while their electrochemical reduction can be enhanced by metal-containing species coupled with carbonized PPy or PANI frameworks. The carbon phase supports electron transport, while the metal or inorganic component promotes catalytic reduction. Metal oxide/carbon interfaces are important to catalytic sensing [201]. During polymer carbonization, for example, Ni-containing precursors can be transformed into NiO/carbon structures. These hybrid interfaces can improve non-enzymatic glucose sensing. The metal oxide phase provides catalytic activity, while the carbon framework improves conductivity and structural support [202]. Metal-containing templates of MOF-derived structures can also improve sensing performance: the inorganic component can provide enrichment or catalytic sites, while the conjugated-polymer-derived carbon layer provides conductivity and interfacial stability. This strategy has been applied to targets like tert–butylhydroquinone, and other electroactive food-related compounds.

Taking Qu et al. (2022) as an example, Figure 11a shows that PPy was synthesized using FeCl_3_ as the oxidizing agent. After carbonization, iron species were retained in the collected carbon materials, because FeCl_3_ was present in the polymer precursor used for pyrolysis [203]. These retained iron-containing species appear to support the electrocatalytic activity of the carbon material in electrochemical sensing; however, the article did not include a metal-free carbon control material; hence, there is no direct comparison to clearly indicate how strongly the iron species contributed to the sensing performance. On the other hand, Jović et al. (2017) introduced 12–phosphotungstic acid during the synthesis of PANI-based materials, thereby generating W-containing species dispersed in the carbonized polyaniline composite, as shown in Figure 11b [204]. As phosphotungstic acid did not play the role of a polymerization oxidant, different concentrations of the metal-containing component could be investigated to study their influence on the properties and electrochemical performance of the material. The study reported that the presence of P and W species from phosphotungstic acid contributed to the sensing performance, enabling a low detection limit for 4–nitrophenol. Wang et al. (2025) approached carbon production through a different method; Figure 11c shows that the authors used MOF−545, which is composed of zirconium ions coordinated with porphyrin ligands, as a template for the formation of a PANI coating over the MOF material [205]. After carbonization, MOF−545@PANI-derived carbon nanomaterials were obtained. The combination of MOF−545 and PANI-derived carbon resulted in a calculated adsorption energy of −1.225 eV, which was stronger than that of the MOF−545 alone of −0.844 eV. Due to the improved electrochemical properties and enrichment capability, the resulting sensor demonstrated strong detection performance and high sensitivity toward tert–butylhydroquinone, a synthetic phenolic antioxidant widely used in food products and edible oils.

Using the work of Jović et al. (2016) as a starting example, Figure 11d shows that carbonized PANI was physically mixed with zeolite materials to improve electrochemical sensing performance, which shows that metal-containing or inorganic materials can still be combined with conjugated-polymer-derived carbon post-carbonization [206]. The resulting composite exhibited better sensing performance than each individual component. However, this method may lead to weaker interactions between the carbon and inorganic components, especially because the decoration or mixing is performed after carbonization. Therefore, although post-carbonization mixing is simple, it may provide less structural integration than pre-carbonization incorporation. Jia et al. (2021) provide another example, in which the concept is similar to that of Wang et al. discussed above, in which PANI was polymerized on an MOF material [207]. However, Figure 11e shows that Jia et al. used a Ni-based MOF. The main difference is that, during PANI pyrolysis, the Ni–MOF was not preserved, but according to the authors, was transformed into NiO; nevertheless, the stronger interaction between NiO and the PANI-derived carbon contributed considerably to the excellent glucose sensing performance. This result suggests that, even when the original MOF structure is not retained, the in situ transformation of the metal-containing precursor can still generate active metal oxide/carbon interfaces for electrochemical sensing. Figure 11f shows the interaction between polymer-derived nitrogen-doped graphene quantum dots and copper nanoclusters in Saisree et al. (2022), which differed from the previous examples [208]. Although both precursors were pre-synthesized, the copper nanoclusters were immobilized on nitrogen-doped graphene quantum dots through coordination and interfacial interactions involving oxygen- and nitrogen-containing surface groups. This system can therefore be considered a stable metal nanocluster/quantum dot hybrid with strong interfacial interaction. In consequence, the material showed excellent simultaneous electrochemical sensing performance toward multiple targets that included dopamine, serotonin, and nicotine.

Across these systems, sensing performance can be broadly related to four coupled processes: analyte adsorption or preconcentration at surface-active sites, electron transfer through the sp^2^-rich carbon framework, mass transport through accessible pores and nanostructures, and, when present, electrocatalytic conversion at metal or inorganic active sites. Because carbonization and surface modification can simultaneously alter several of these factors, the contribution of an individual structural parameter cannot always be isolated. Representative analytical figures of merit and the dominant material-level sensing mechanisms are therefore summarized in Table 2.

## 7. Challenges and Perspectives

Conjugated-polymer-derived carbon nanomaterials offer considerable potential for electrochemical sensing, but several precursor-specific challenges remain. First, the relationship between the initial polymer structure and the resulting carbon is still insufficiently understood. The oxidation/doping state, counterions, molecular organization, and morphology of conjugated polymers can strongly influence carbonization, while intrinsic heteroatoms such as N in PPy/PANI and S in PT/PEDOT may be removed, redistributed, or converted into different chemical configurations during pyrolysis. Consequently, similar polymer precursors can produce substantially different conductivity, defect density, porosity, and surface chemistry depending on their initial state and carbonization conditions. Establishing clearer precursor–carbon structure–sensing performance relationships is therefore essential for rational material design.

Another important issue is distinguishing the true origin of electrochemical activity. High heteroatom content alone does not guarantee high sensing performance because the configuration and surface accessibility of heteroatoms are often more important than their total concentration. Furthermore, oxidants, dopant counterions, templates, or metal species introduced during conjugated-polymer synthesis may remain or transform during carbonization and contribute to the observed electrochemical response. Morphology inheritance also requires greater attention: although conjugated polymers can be readily prepared as nanotubes, hollow structures, fibers, and porous architectures, thermal shrinkage and densification may alter or partially destroy these features during carbonization. Future studies should therefore emphasize controlled precursor synthesis, systematic tracking of heteroatom and morphology evolution, appropriate control samples, and standardized carbonization conditions. These efforts will help translate the structural versatility of conjugated polymers into reproducible and application-oriented carbon sensing materials.

## 8. Conclusions

The literature comparison indicates that sensing performance is governed less by any single structural parameter than by how precursor chemistry, carbonization, and interfacial structure are coupled. Conjugated polymers should therefore be selected according to the type and accessibility of heteroatom sites expected after carbonization, rather than simply by their initial elemental content. PPy and PANI are advantageous when N-containing surface sites are desired, whereas PT and PEDOT provide a route to S-containing carbons with different electronic and adsorption characteristics. In both cases, the retained heteroatom configuration and its accessibility to the analyte are more relevant than the total heteroatom concentration.

Carbonization likewise requires a compromise between electrical conductivity and chemical activity. Increasing structural ordering can improve electron transport, but excessive treatment may remove heteroatoms and reduce defect-rich surface sites. The most effective sensing materials therefore tend to retain sufficient disorder and surface functionality while maintaining a conductive carbon framework. Morphology should also be considered after, rather than only before, carbonization, because shrinkage, densification, and pore collapse can substantially change the accessible surface and mass transport pathways initially created in the polymer precursor.

These relationships also clarify the role of additional dopants and metal species. Their incorporation is most useful when it produces a specific function—such as improved analyte adsorption, catalytic conversion, or interfacial charge transfer—rather than simply increasing compositional complexity. Taken together, the literature suggests that rational design of conjugated-polymer-derived sensing carbons should focus on preserving accessible active sites while simultaneously maintaining efficient electron and mass transport. This precursor-to-carbon perspective provides a more useful basis for material selection than optimizing heteroatom content, surface area, or metal loading independently.

## Figures and Tables

**Table 1:** Carbonization conditions and structural characteristics of representative conjugated-polymer-derived carbon materials.

Conjugated Polymer Precursor	Template/Dopant/Precursor Morphology	Carbonization/Activation	Atmosphere	BET ^1^ Surface Area (m^2^ g^−1^)	Heteroatom Content/Chemical States	Resulting Carbon Morphology/Structure	Ref.
PANI	Granular PANI base	Heating up to 800 °C; ~650 °C/1 h identified as favorable carbonization condition	Inert	NR	N retained in carbon; N content decreases with increasing treatment severity	Granular morphology largely retained; N-containing carbon	[55]
PANI	Colloidal PANI nanoparticles stabilized with PVP ^2^	650 °C	Inert	~200	Up to 10.8 wt% N; C/N atomic ratio ~7–8	Carbon nanoparticles with precursors morphology retained	[101]
	Colloidal PANI nanoparticles stabilized with silica	650 °C	Inert	~205	N-containing carbon	Nanoparticulate morphology retained after carbonization	
PANI	Self-assembled PANI nanotubes	Heating to ~830 °C	N_2_	NR	N-containing carbon; thermal conversion modifies N configurations	Carbon nanotubes, with tubular precursor morphology substantially retained	[102]
PANI	PANI micro-/nanotubes	Carbonization studied to 1100 °C; strong carbonization at ≥800 °C	Inert	NR	N progressively lost with increasing temperature	Highly carbonized micro-/nanotubes; morphology remains recognizable	[16]
PANI	H_2_SO_4_-protonated PANI nanotubes	800 °C, 10 °C min^−1^	N_2_	NR	~9 wt% N; N remains incorporated into carbon network	Conducting carbonized nanotubes	[103]
PANI-SSA ^3^	5-Sulfosalicylic-acid-doped PANI nanorods/nanotubes	800 °C	N_2_	~317	Approximately 10 wt% N retained	Micro/mesoporous carbonized nanorods/nanotubes	[104]
PANI-DNSA ^4^	3,5-Dinitrosalicylic-acid-doped PANI nanorods	Gradual heating to 800 °C	N_2_	~441	9.8 wt% N; multiple N configurations observed by XPS ^5^	Microporous N-containing carbon nanorods	[105]
Nano-PANI	Sol–gel-derived nano-PANI	1000 °C	N_2_	14.42	N-containing carbon; exact surface speciation NR	Carbonized nanoscale particles; precursor morphology substantially preserved	[106]
PANI	Chemically synthesized PANI	800 °C; residence time varied	Inert	NR	N content/C:N ratio strongly dependent on carbonization duration	PANI-derived N-doped carbon	[59]
PPy	Tubular PPy precursor	Carbonization followed by porosity development/activation	Inert	~1765	N-doped carbon; pyridinic/pyrrolic/graphitic-type N present	Porous N-doped carbon nanotubes; inner diameter ~55 nm, wall ~22 nm	[56]
PPy	PPy + KOH chemical activation	600–800 °C	Inert	~1700 under mild 600 °C activation	Up to ~10 wt% N at milder activation; N decreases at higher severity	Highly microporous N-doped carbon	[107]
PPy	PPy-derived carbon followed by steam activation	Pyrolysis at ~900 °C, followed by steam activation	Inert → steam	Strongly increased by steam activation	N functionalities progressively decrease during activation	N-doped activated porous carbon	[58]
PPy	Globules, nanofibers and nanotubes	100–700 °C	Ar	Morphology-dependent	N retained but progressively transformed/lost during heating	Carbon morphology strongly inherits initial PPy morphology	[64]
PPy	Pyrolyzed PPy	Temperature-dependent pyrolysis	Inert	NR	Pyrrolic, pyridinic and graphitic/quaternary N evolve systematically with temperature	N-containing carbonaceous framework	[65]
PPy	Methyl-orange-assisted PPy nanotubes; FeCl_3_ oxidant	Carbonization of PPy nanotubes	Inert	NR	N-containing carbon; pyridinic/pyrrolic-type surface N	Mesoporous PPy-derived carbon nanotubes, tubular structure preserved	[108]
PT	Sulfur-rich polymeric carbon precursor; activation	Carbonization + activation	Inert/activating treatment	Up to ~2000 m^2^ g^−1^ class depending on treatment	S-doped carbon; residual S decreases as activation severity increases	Highly porous S-doped carbon	[57]
N/S-containing conjugated polymers	N- and S-containing polymer precursors	Carbonization + activation	Inert/activating treatment	Treatment-dependent	N-, S- and N/S-doped surfaces	Heteroatom-doped activated porous carbons	[54]
N/S-containing conjugated polymer system	Conjugated-polymer-templated precursor	Controlled carbonization	Inert	Treatment-dependent	N,S co-doped carbon; heteroatom ratio tunable through precursor design	Tunable porous N,S-co-doped carbon structure	[53]

^1^ BET: Brunauer–Emmett–Teller method; ^2^ PVP: polyvinylpyrrolidone; ^3^ SSA: 5-sulfosalicylic acid; ^4^ DNSA: 3,5-dinitrosalicylic acid; ^5^ XPS: X-ray Photoelectron Spectroscopy.

**Table 2:** Electrochemical sensing performance of representative conjugated-polymer-derived carbon materials.

Polymer Precursor	Derived Carbon/Composite	Target Analyte	Electrode/Method	Linear Range	LOD ^1^	Sensitivity/Key Response	Real Sample/Practical Test	Ref.
PANI	MnO_2_/carbonized nanostructured PANI	H_2_O_2_	Modified electrode; voltammetric electroanalysis	NR	NR	High electrocatalytic activity toward H_2_O_2_	Aqueous medium	[209]
PANI	PANI-derived N-doped carbon	Ascorbic acid	Carbon-modified electrode; CV ^2^	NR	NR	Carbonization considerably enhances AA electrooxidation relative to precursor	NR	[59]
PANI	N-doped carbon nanorods/Nafion	Dopamine	GCE ^3^; DPV ^4^/CV	0.008–15 µM	8.9 nM	Strong discrimination of DA in presence of excess AA	NR	[60]
PANI, PANI-SSA ^5^, PANI-DNSA ^6^	Carbonized nanostructured PANIs	Nitrite; ascorbic acid	GC ^7^/carbonized PANI; LSV ^8^/CV	NR	NR	Lower oxidation overpotential and enhanced oxidation current; performance depends on precursor dopant	Aqueous analysis	[177]
PANI	PANI-derived N-doped graphene quantum dots	2,4,6-Trinitrophenol and nitroaromatics	N-GQD ^9^/GCE; voltammetric detection	NR	~0.2 ppb (~nM level)	Ultra trace detection, electrochemical differentiation of structurally related nitroaromatics	Water samples	[181]
PANI	PANI-derived N-doped porous carbon + glucose oxidase	Glucose	Enzyme/N-carbon electrode; amperometric O_2_-consumption route	5 µM–5 mM	NR	23.57 ± 1.77 µA mM^−1^ cm^−2^	Human urine; commercial sugary drink; AA ^10^/DA ^11^/UA ^12^ showed negligible interference	[210]
PANI	PANI-derived N-GQDs	Cd(II)	N-GQD/GCE; voltammetric detection	Very broad LDR ^13^ reported	Down to 10^−15^ M with pre-reduction	~3.57 µA µM^−1^ cm^−2^	Environmental water samples; good reusability/selectivity	[182]
PANI hydrogel	3D HPG ^14^ carbon	Sunset Yellow	HPG/GCE; voltammetric sensor	Broad range reported	0.15 nM	5285.7 A M^−1^ cm^−2^	Beverage/food analysis	[211]
PANI hydrogel	N,O-co-doped 3D hierarchical porous graphitic carbon	*Lactobacillus rhamnosus* GG	Label-free electrochemical immunosensor	Linear response; R^2^ = 0.9976	2 CFU ^15^ mL^−1^	BET 4859 m^2^ g^−1^ provides very high antibody-loading/interface area	Dairy products and drinks; good specificity and long-term stability	[212]
PPy	PPy-derived carbon nanotubes/PEI ^16^/AuNPs	Caffeine	Hybrid/GCE; CV/DPV	10 nM–10 mM	2.8 nM	Carbonized-CNT hybrid response ≈ 4.5 × higher than corresponding nanotube system	Beverage-related samples; good reproducibility/interference resistance	[180]
PPy	Hierarchical PPy-derived porous carbon nanosheets/Fe_3_O_4_	Catechin	mNPC/Fe_3_O_4_/GCE; DPV	0.1 nM–1.1 µM	0.36 nM	High response attributed to hierarchical porosity + conductive carbon + Fe_3_O_4_ catalytic sites	Complex sample matrices; strong anti-interference performance	[178]

^1^ LOD: limit of detection; ^2^ CV: cyclic voltammetry; ^3^ GCE: glassy carbon electrode; ^4^ DPV: differential pulse voltammetry; ^5^ SSA: 5-sulfosalicylic acid; ^6^ DNSA: 3,5-dinitrosalicylic acid; ^7^ GC: glassy carbon; ^8^ LSV: linear sweep voltammetry; ^9^ GQD: graphene quantum dot; ^10^ AA: ascorbic acid; ^11^ DA: dopamine; ^12^ UA: uric acid; ^13^ LDR: linear dynamic range; ^14^ HPG: hierarchical porous graphitic; ^15^ CFU: colony-forming unit; ^16^ PEI: polyethyleneimine.

## Data Availability

No new data were created or analyzed in this study. Data sharing is not applicable to this article.

## Abbreviations

The following abbreviations are used in this manuscript:

Ppy	Polypyrrole
PANI	Polyaniline
EDOT	3,4-ethylenedioxythiophene
PEDOT	Poly (3,4-ethylenedioxythiophene)
PT	Polythiophene
